# Next-generation intranasal influenza vaccines: mechanisms, platforms, and translational progress

**DOI:** 10.3389/fimmu.2026.1809302

**Published:** 2026-04-20

**Authors:** Laise Rodrigues Reis, Ted M. Ross

**Affiliations:** 1 Florida Research and Innovation Center, Cleveland Clinic, Port Saint Lucie, FL, United States; 2 Department of Infection Biology, Lerner Research Institute, Cleveland Clinic, Cleveland, OH, United States; 3 Department of Infectious Diseases, University of Georgia, Athens, GA, United States; 4 Center for Vaccines and Immunology, University of Georgia, Athens, GA, United States

**Keywords:** flu vaccine, influenza, influenza vaccine, intranasal vaccine, mucosal immunity

## Abstract

Seasonal influenza vaccination remains the most effective strategy for reducing influenza burden and preventing severe disease. Despite decades of vaccine development, the seasonal influenza vaccine is administered intramuscularly and provides suboptimal and highly variable effectiveness depending on host factors, pre-existing immunity, and antigenic match between vaccine and circulating strains. Recent advances in vaccine development have highlighted the potential of intranasal vaccine delivery as a strategy to increase protection against influenza virus infection by inducing local and systemic immune responses. Across multiple intranasal platforms under development, mucosal immunity, particularly secretory IgA and T- and B-cell immune responses, plays a central role in shaping protection against influenza virus infection. Live-attenuated influenza vaccines (LAIV) elicit protective immune responses, particularly in the pediatric population, and remain the only currently licensed intranasal seasonal influenza vaccine. However, variable performance in adults, strain-dependent viral fitness, and clinical contraindications have limited their broader applicability. These limitations have driven the development of next-generation intranasal influenza vaccine platforms designed to preserve the immunological advantages of mucosal vaccination while improving consistency, safety, and applicability across diverse populations. This review synthesizes current knowledge on licensed and emerging intranasal influenza vaccine platforms, including replicating viral platforms and non-replicating platforms, and discusses key immunological mechanisms, challenges, and translational progress. Together, these advances underscore the growing potential of intranasal vaccination as a next-generation strategy to improve influenza control.

## 1 Introduction

Influenza viruses continue to pose a major global health and socioeconomic burden, causing an estimated 3–5 million severe infections and up to 650,000 deaths each year worldwide (1, 2). Seasonal influenza vaccination remains the most effective strategy for reducing this burden and preventing severe disease. Currently licensed influenza vaccines are produced using egg-, cell-, or recombinant protein-based platforms, with intramuscular (IM) formulations representing the predominant route of administration worldwide (3, 4). These vaccines primarily induce humoral responses targeting the highly variable surface glycoproteins, hemagglutinin (HA) and, to a lesser extent, neuraminidase (NA) (5, 6). HA-directed antibodies play a central role in protection by preventing viral attachment and entry into host cells, thereby mediating neutralization (7, 8). However, strong immune pressure on immunodominant epitopes located within the HA head region drives continuous antigenic drift, allowing the virus to escape pre-existing immunity and necessitating annual vaccine reformulation to match circulating strains, including the H1N1 and H3N2 subtypes of influenza A (IAV) and the Victoria lineages of influenza B (IBV) (3, 9–12). Despite decades of vaccine development, the seasonal influenza vaccine still provides suboptimal and highly variable effectiveness, ranging from 30 to 60% depending on host factors, pre-existing immunity, and antigenic match between vaccine and circulating strains (12–19).

An important limitation of IM vaccines is their limited ability to elicit immune responses at the primary site of infection, the respiratory mucosa. The respiratory mucosa represents the first line of defense against respiratory diseases, where secretory IgA, along with tissue-resident memory T- and B-cells, is thought to provide rapid and localized protection (20–25). Together, these components are associated with limiting viral replication and shedding and may contribute to reduced transmission and enhanced cross-protective immune responses against heterologous or drifted strains (23, 24, 26). Due to their predominant induction of systemic responses rather than mucosal immune responses, IM vaccines are less effective at rapidly preventing viral entry and transmission and provide limited cross-protective immunity against different influenza strains. These limitations emphasize the need for next-generation vaccine strategies that can induce both systemic and mucosal immune responses.

Recent advances in vaccine development have highlighted the potential of intranasal vaccines as a strategy to increase protection against influenza and overcome these limitations by inducing local and systemic immune responses (27). Continued efforts in vaccine formulation, including the use of adjuvants, viral vectors, and delivery platforms, have further improved the immunogenicity of intranasal vaccines (27–29). Besides the immune mechanisms that collectively help block early viral replication, reduce shedding, and provide broader protection, intranasal vaccines offer practical and functional advantages over traditional IM vaccines. One of the benefits is the reduction of risks associated with needles, such as potential transmission of blood-borne diseases, including HIV and hepatitis B (30). In addition, intranasal delivery increases patient compliance, particularly among children, by avoiding pain and needle anxiety, factors that often contribute to vaccine hesitancy (31, 32). The possibility of self-administration further supports its use in large-scale immunization campaigns, enabling rapid distribution during outbreaks or pandemics (30). From a logistical perspective, intranasal vaccines may also reduce production and distribution costs, as they can be formulated in both liquid and dry forms, which are less dependent on cold-chain maintenance and more stable under temperature fluctuations (30). Together, these advances highlight intranasal vaccination as a promising next-generation approach capable of inducing both long-lasting and cross-protective immunity, addressing key limitations of conventional IM influenza vaccines.

Based on the advantages of intranasal vaccination and its potential as a next-generation strategy, this review provides a comprehensive overview of the current landscape of intranasal influenza vaccines. It highlights recent advances in vaccine formulation, delivery platforms, and adjuvant strategies, while exploring immunological mechanisms underlying mucosal protection and offering insights to guide future research and development in the field.

## 2 Immunological basis of intranasal influenza vaccination

**Figure 1:**
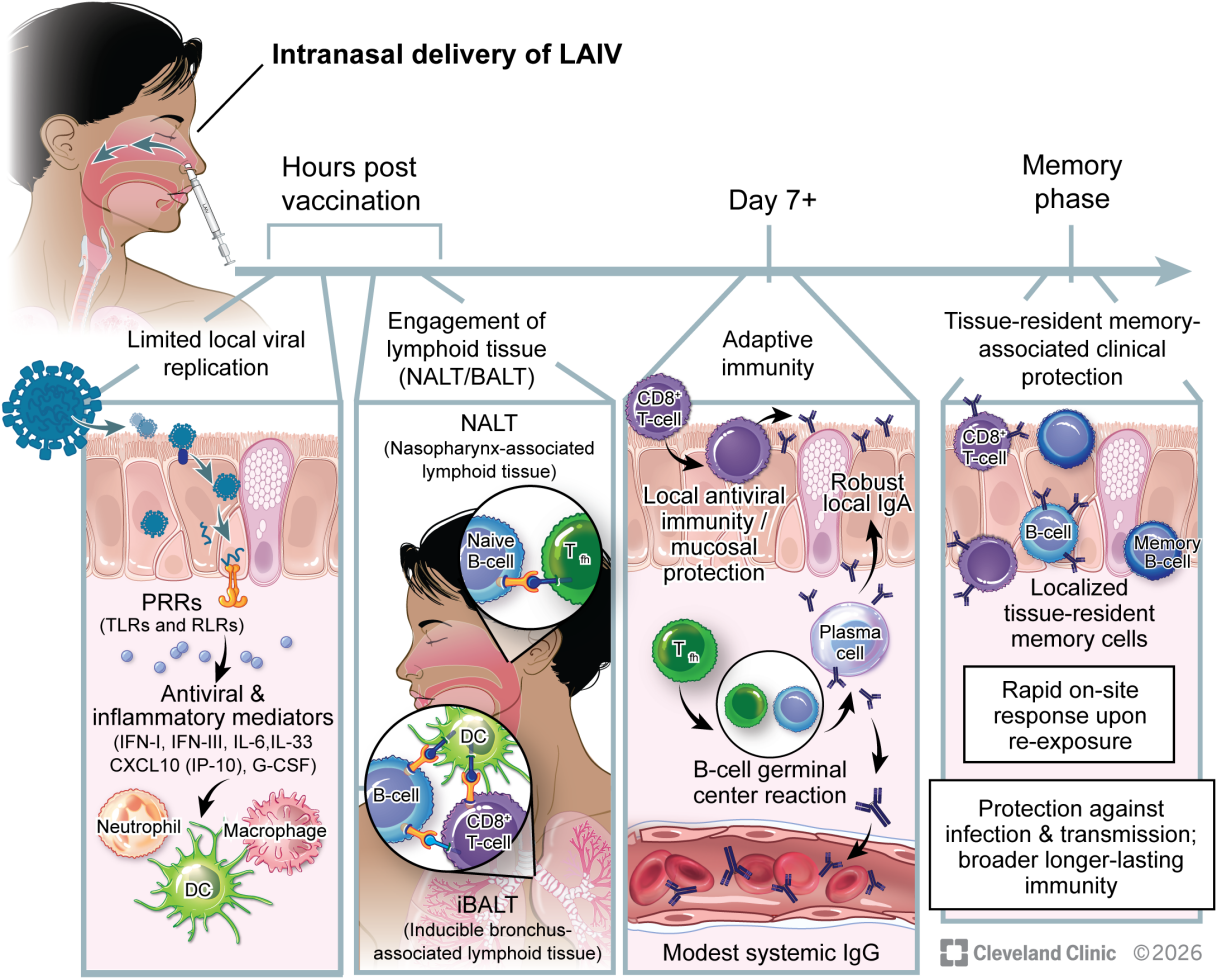
Immunological basis of live-attenuated influenza vaccine (LAIV). LAIV, the only intranasal influenza vaccine currently licensed for human use, targets the mucosa of the upper respiratory tract (URT) and initiates limited local viral replication, leading to rapid activation of innate immune pathways within hours post-vaccination. Early events include epithelial sensing through pattern-recognition receptors (PRRs), such as Toll-like receptors (TLRs) and RIG-I-like receptors (RLRs), leading to the production of antiviral and inflammatory mediators, establishment of an antiviral mucosal environment, and recruitment of innate immune cells. Intranasal vaccination also promotes engagement of mucosal lymphoid structures, including nasopharynx-associated lymphoid tissue (NALT) and inducible bronchus-associated lymphoid tissue (iBALT), which function as local sites of antigen presentation, lymphocyte activation, and B-cell differentiation. Within iBALT, dendritic cells support local activation and reactivation of CD8^+^ T cells and facilitate antigen availability for B cells, while B-cell maturation and class switching occur in coordination with CD4^+^ T-cell help. From approximately day 7 post-vaccination, adaptive immune responses are established, characterized by activation of CD8^+^ T-cells and the expansion of T follicular helper (Tfh) cells, leading to robust mucosal IgA response alongside a modest systemic IgG response. During the memory phase, intranasal vaccination can promote the establishment of tissue-resident cells within the respiratory mucosa, enabling rapid on-site immune responses upon re-exposure and contributing to clinical protection.

Respiratory mucosal immunity plays a central role in protecting against respiratory pathogens, acting as the first line of defense and providing a multifaceted immune response (33, 34). Inducing immunity directly at the site of infection is an effective strategy for limiting viral replication, reducing transmission, and enhancing vaccine-mediated protection (35, 36). Intranasal influenza vaccination, delivered as a nasal spray, targets the mucosa of the upper respiratory tract (URT), activating immune pathways that are not efficiently elicited by conventional IM vaccines (34, 37–39). Following intranasal administration of a live-attenuated influenza virus vaccine (LAIV), the immune response closely mimics natural infection in the URT (**Figure 1**). The attenuated virus undergoes limited replication in epithelial cells, activating innate immune pathways through pattern-recognition receptors, including Toll-like receptors (TLRs) and RIG-I–like receptors (RLRs) (40, 41). This recognition induces type I and III interferons and pro-inflammatory cytokines, establishing an early antiviral environment in the mucosa (42–44). Although much of this mechanistic understanding has been described in the context of wild-type influenza infection, these pathways are thought to also inform immune recognition of attenuated vaccine strains.

Human LAIV studies provide direct evidence of these pathways. In a controlled intranasal LAIV study in healthy adults, there were increased levels of IL-33 in the nasal fluid within 8 hours post-inoculation followed by a dominant interferon-led transcriptional program over the next 24–72 hours, including increased levels of CXCL10, IL-6, IFN-γ and type III interferon (25). LAIV elicits a localized inflammatory response, characterized by increased levels of IL-6, IP-10, and G-CSF, despite minimal systemic cytokine changes (45). Together, these findings indicate that LAIV rapidly recruits and activates local innate immune cells in the URT, establishing a strong antiviral environment in the mucosa and creating the conditions that precede the adaptive immune response.

A defining feature of intranasal vaccination is its engagement of nasal-associated lymphoid tissues (NALT), particularly the adenoids and tonsils of Waldeyer’s ring, which serve as sites for early T follicular helper (Tfh) and B-cell priming. LAIV administration activates tonsillar Tfh and CD8^+^ T-cells in adults and children, highlighting the importance of these mucosal lymphoid tissues in orchestrating adaptive immune responses (46, 47). Additionally, influenza virus infection stimulates bronchus-associated lymphoid tissue (iBALT), which has been shown to support local antigen presentation and may provide a niche for memory T-cell priming and B-cell maturation (48–50). Whether currently licensed LAIV formulations reliably induce iBALT remains unknown, these models provide a mechanistic rationale for how intranasal vaccines may generate durable and tissue-localized memory.

By approximately day 7 post-vaccination, the adaptive immune response is detectable with evidence of CD8^+^ T-cell activation and expansion of circulating Tfh cells (25, 46, 47). These cellular responses are associated with distinct humoral outcomes, with CD8^+^ T-cell activation associated with a stronger mucosal IgA response, whereas greater expansion of Tfh cells is related to systemic IgG response (25). Consistent with this, LAIV preferentially induces increased levels of mucosal IgA with modest serum IgG responses (45). Approximately 9% of LAIV vaccinated participants had serum HAI seroconversion, whereas ~33% of these participants had at least a two-fold rise in HA-specific IgA in nasal wash (45), supporting the idea that intranasal vaccination generates both mucosal and systemic responses, but a more robust mucosal immune response (25, 47, 51). Human challenge studies further support the protective role of mucosal IgA, showing that nasal IgA in both children and adults correlates with reduced influenza infection (52–54). Moreover, studies assessing seasonal LAIV immunization in children describe long-lasting and broader clinical protection, supporting the multifaceted LAIV-mediated immune response, including mucosal IgA, systemic IgG, and T- and B-cell responses (55–57).

Importantly, these features are also associated with enhanced cross-protective immunity against antigenically drifted influenza strain, a major limitation of current IM vaccines. Mucosal IgA responses exhibit broader reactivity due to increased avidity compared to IgG (58, 59) and have been associated with cross-reactivity across influenza A subtypes (60, 61), while cross-reactive CD8^+^ T cells target conserved internal viral proteins that are less susceptible to antigenic drift (62, 63). In addition, tissue-resident memory responses enable rapid viral control at the site of infection, limiting viral replication even when neutralizing antibodies are supoptimal or mismatched (64, 65). Consistent with this, both human and preclinical studies have shown that intranasal vaccination can provide protection against heterologous influenza strains, supporting its potential to offer broader and more durable immune responses in the context of ongoing viral evolution.

The kinetics of antigen exposure also contribute to these immunological distinctions. LAIV replicates locally in the URT and can be detected for several days after administration (66, 67), providing longer antigen availability compared to non-replicating vaccines. Although the full impact of this sustained antigen availability on Tfh activation and mucosal antibody maturation has not been directly defined for LAIV, prolonged antigen availability has been shown to enhance germinal-center responses, Tfh engagement, affinity maturation, and antibody responses (68–70). Building on this framework, IN vaccination may favor the establishment of immune memory within the URT.

Despite limited evidence of nasal tissue-resident memory T- and B-cells in humans following LAIV administration, converging data from human challenge studies and translational models indicate that LAIV may contribute to the establishment of tissue-resident memory within the URT. Human challenge studies demonstrate that pre-existing mucosal immunity, particularly nasal IgA, and cross-reactive CD8^+^ T-cell responses measured in blood, correlate with protection independently of systemic antibody titers (38, 53, 65). Although these studies do not directly evaluate tissue-resident populations, they support the broader principle that protection is shaped not only by systemic antibodies, but also by localized and cellular immune responses. Complementing this, translational studies in murine influenza models further demonstrate that mucosal immunization robustly elicits lung resident memory CD8^+^ T-cells, capable of rapid viral control and cross-protection (71, 72), responses that are not reproduced by IM vaccination. Preclinical mechanistic studies further demonstrate that influenza virus infection establishes durable tissue-resident memory B-cells in the lung, which possess distinct transcriptional profiles and rapidly differentiate into antibody-secreting cells upon rechallenge (73–75).

Taken together, these findings show that intranasal influenza vaccination uses the natural biology of the URT to build a multilayered immune response. By combining local viral replication, rapid interferon-driven activation, sustained antigen exposure, and the engagement of specialized mucosal lymphoid tissues, LAIV shapes immune responses that are difficult to achieve through IM immunization. This approach not only strengthens mucosal IgA responses and primes T- and B-cells directly at the site of viral entry but also may support the activation of tissue-resident memory capable of responding within moments after exposure. The result is an immune profile that is broader, more rapid, and may be more effective at limiting infection and potentially transmission than what is typically achieved with IM inactivated vaccines, underscoring the unique advantages of intranasal vaccination over traditional IM approaches.

## 3 Current and developmental intranasal influenza vaccine platforms

Intranasal influenza vaccine development has progressed along distinct technological trajectories. In contrast to IM influenza vaccines, which primarily elicit systemic immune responses, intranasal platforms are designed to target the URT, the site of influenza virus entry and early replication. By engaging local innate and adaptive immune pathways, intranasal vaccination has the potential to limit viral replication and reduce transmission. However, translating these immunological advantages into consistent clinical performance has required consistent vaccine optimization, including safety, immunogenicity, and delivery efficiency in the nasal mucosa. In this context, current intranasal influenza vaccine strategies include both replicating viral platforms and non-replicating platforms (**Table 1**). Each approach employs distinct mechanisms to induce mucosal and systemic immune responses.

**Table 1:** Current and developmental intranasal influenza vaccine platforms.

Platform	Replication	Key features	Immune response	Stage of development	Key References
LAIV	Replicating (multi-cycle)	Cold-adapted, temperature-sensitive live-attenuated viruses optimized for URT replication	Mucosal IgA, serum IgG, HAI titers, neutralizing antibodies, and CD4^+^/CD8^+^ T-cell responses.	Licensed / Approved	(76, 77, 79, 83)
M2SR	Replicating (single-cycle)	M2-deficient influenza viruses that do not generate infectious virus progeny	Mucosal IgA, serum IgG, neutralizing antibodies, and CD4^+^/CD8^+^ T-cell responses.	Phase II	(94, 97, 98, 101)
PIV5 vector	Replicating (multi-cycle)	Non-segmented paramyxovirus vector with no known association with human disease	Mucosal IgA, serum IgG, neutralizing antibodies, CD4^+^/CD8^+^ T-cell responses.	Preclinical	(102–104, 107)
Ad5 vector	Non-replicating	Replication-deficient adenovirus serotype 5 vector	Mucosal IgA, serum IgG, neutralizing antibodies, and T−cell responses.	Phase II	(113–116)
Adjuvanted protein-based	Non-replicating	Purified or recombinant viral antigens formulated with mucosal adjuvants	Mucosal IgA, serum IgG, neutralizing antibodies, CD4^+^/CD8^+^ T-cell responses, memory B-cell responses.	Preclinical / Phase I	(123, 124, 126, 131)
VLP-based	Non-replicating	Self−assembling nanoparticles mimicking virion structure without viral genetic material	Mucosal IgA, serum IgG, neutralizing antibodies, CD4^+^/CD8^+^ T-cell responses.	Preclinical	(132, 136, 137, 140)

### 3.1 Replicating intranasal platforms

#### 3.1.1 LAIV

LAIV are the only intranasally administered influenza vaccines currently licensed for human use, providing the most extensive clinical evidence supporting the prevention of influenza virus infection (57). These vaccines are based on cold-adapted, temperature-sensitive influenza viruses that replicate efficiently at the cooler temperatures of the URT, while remaining restricted at higher temperatures encountered in the lower respiratory tract (76, 77). This localized replication elicits a multifaceted compartmentalized immune response (25) that differs from that induced by IM inactivated vaccines. LAIV robustly induce mucosal secretory IgA in the URT, which can persist for months in both children and adults (78, 79). Systemic humoral responses, including serum IgG and hemagglutination inhibition (HAI) titers, are also generated, although these are typically of lower magnitude than those elicited by IM inactivated vaccines (80–82). In addition, LAIV induces influenza virus-specific CD4^+^ and CD8^+^ T cell responses, characterized by IFN-γ production, proliferative capacity, and polyfunctional cytokine profiles (78, 81). These cellular responses are generally more pronounced in children, likely reflecting reduced pre-existing immunity and greater vaccine virus replication, and have been associated with broader cross-protection against antigenically diverse influenza strains (78, 79, 83).

Consistent with these immunological features, evidence supporting the clinical efficacy of LAIV has been more consistently reported in pediatric populations. Pivotal trials showed that LAIV conferred superior protection compared to IM inactivated influenza vaccines, particularly against A/H3N2 and influenza B strains, reflecting broader and more durable protection in this age group (84–87). Additionally, pooled analysis and meta-analyses have reinforced these findings, reporting comparable or superior efficacy of LAIV to IM inactivated vaccines in preventing laboratory-confirmed influenza virus infection in children (85, 88). These outcomes are attributed to the limited pre-existing immunity in children, which allows more efficient replication of the attenuated vaccine virus in the URT (79, 85). This promotes robust engagement of mucosal immune pathways, including innate immune activation, mucosal IgA induction, and cellular immune priming, which together likely contribute to broader and more durable protection, including partial cross-protection against antigenically divergent strains (78, 79, 85).

In contrast, vaccine effectiveness in adults has varied considerably across influenza seasons, particularly in comparative studies of LAIV and IM inactivated vaccines, where LAIV has shown comparable to or lower protection against laboratory-confirmed influenza than that conferred by IM inactivated vaccines in healthy adults (89, 90). This reduced effectiveness has been attributed to pre-existing anti-influenza virus immunity that may limit vaccine virus replication in the URT, as well as strain-specific differences in viral fitness and potential interference among vaccine strains (91–93). These findings indicate that the protective efficacy elicited by LAIV is closely dependent on its capacity for local replication and subsequent activation of mucosal immune responses, underscoring fundamental age-dependent differences in vaccine performance.

Consistent with these age-dependent differences in clinical effectiveness, LAIV vaccination is approved and recommended for healthy, non-pregnant individuals aged 2–49 years, while its use is not advised in adults ≥50 years, immunocompromised individuals, pregnant women, or those with specific underlying conditions (3).

#### 3.1.2 M2-deficient single replication vaccine virus

M2SR influenza vaccines represent an investigational, next-generation class of replicating intranasal vaccine platforms based on single-cycle viruses. These vaccine viruses can infect host cells and elicit robust humoral and cell-mediated immune responses without generating infectious virus progeny (94). M2SR vaccines are designed to lack the matrix protein 2 (M2), an ion channel essential for viral uncoating and completion of the replication cycle (94–96). As a result, the vaccine viruses can enter host cells and express all viral proteins except M2, while remaining unable to produce infectious progeny, thereby restricting infection to a single replication cycle (94, 97).

This replication-restricted strategy allows effective intracellular antigen expression and presentation, closely mimicking the early stages of natural infection, while limiting the risks associated with virus shedding and potential reassortment (94, 98, 99). These features make M2SR particularly attractive for populations in which LAIV efficacy is reduced or contraindicated, such as adults with pre-existing immunity, older adults, and immunocompromised individuals.

Preclinical studies in murine and ferret models have shown that intranasal administration of M2SR elicits broad influenza-specific immune responses, including mucosal, humoral, and cellular compartments (94, 99, 100). Specifically, M2SR vaccination elicits robust mucosal IgA in the URT, along with serum IgG responses, and functional neutralizing antibodies measured by microneutralization (MN) assays against both homologous and antigenically drifted strains (94, 99, 100). In addition, M2SR vaccination activates influenza-specific CD4^+^ and CD8^+^ T cell responses, including IFN-γ production, consistent with a cellular immune profile observed in influenza virus natural infection (94, 99).

Clinical evaluation of M2SR vaccines has progressed to phase I/Ib and phase II studies in healthy adults (ClinicalTrials.gov NCT03999554; ClinicalTrials.gov NCT02822105) and demonstrated that intranasal M2SR is well tolerated with an acceptable safety profile and no detection of vaccine virus shedding or transmission (29, 97). Importantly, M2SR elicits systemic and mucosal immune responses even in individuals with pre-existing influenza immunity that demonstrates that antigen expression without multi-cycle replication can induce a robust immune response where LAIV responsiveness is limited (29).

Building on these findings, controlled human influenza challenge studies further support the clinical relevance of M2SR vaccination. In a phase II challenge model using a heterologous A/H3N2 virus (EudraCT no. 2017–004971–30), prior M2SR vaccination was associated with reduced rates of laboratory-confirmed influenza virus infection, decreased viral shedding, and attenuation of clinical illness (101). Although protective efficacy varied among participants, these results highlight the ability of M2SR to induce immune responses that translate into protection against antigenically distinct strains.

Although preclinical and early clinical findings demonstrated encouraging results, important questions remain for the continued development of M2SR vaccines. Future studies are needed to assess the longevity of mucosal and systemic immune responses, the extent of cross-protection against antigenically drifted and heterologous influenza strains, and to identify correlates of protection that best predict clinical protection. Moreover, larger and more diverse clinical trials across different age groups and risk populations are necessary to assess performance and to establish how M2SR compares directly with existing intranasal and IM influenza vaccines. Addressing these aspects is essential to defining the potential role of M2SR within evolving influenza vaccine strategies.

#### 3.1.3 Parainfluenza virus 5

PIV5 is a non-segmented, negative sense RNA virus of the *Paramyxoviridae* family that has been developed as a replicating intranasal vaccine vector. PIV5 efficiently infects respiratory epithelial cells without association with known human disease, making it an attractive platform for mucosal antigen delivery (102). Recombinant PIV5 vectors can be designed to express influenza virus antigens, allowing robust antigen expression in the URT and providing protective immune response while maintaining a safety profile in preclinical models (103–105).

An additional advantage of PIV5-based vaccines is the low to moderate prevalence of pre-existing immunity in humans, which reduces interference from vector-specific immune responses (102, 106). Moreover, due to a non-segmented genome of PIV5, it does not undergo gene segment reassortment (107), thereby reducing safety concerns commonly associated with replication-competent influenza-based vaccine vectors.

Based on these biological and safety features, PIV5-based influenza vaccine candidates have progressed to extensive preclinical evaluation. In animal models, a single intranasal dose of recombinant PIV5 vectors expressing influenza HA elicits protective immune response after challenge with a lethal highly pathogenic avian influenza H5N1 virus (104, 108). In addition, related PIV5−based vaccines expressing H5N1 antigens such as neuraminidase (NA) or nucleoprotein (NP), have elicited substantial cross-protection against homologous and heterologous influenza A viruses (105, 109). The protective response observed in these studies was associated with both humoral and cellular immune responses. The PIV5-based intranasal vaccination elicited HA-specific serum IgG, neutralizing antibodies and mucosal IgA in the URT (104, 108, 109). In addition, PIV5 vectors expressing nucleoprotein induces influenza virus-specific CD8^+^ T-cell responses, characterized by IFN-γ production and cytotoxic activity (105, 109).

Clinical evaluation of PIV5-based influenza vaccines remains limited. However, human studies using this platform for respiratory syncytial virus (RSV) and severe acute respiratory syndrome coronavirus 2 (SARS-CoV-2) vaccines have demonstrated acceptable safety and tolerability, with evidence of both systemic and mucosal immune response following intranasal administration (110, 111). These findings support the translational potential of PIV5 vectors for influenza vaccination.

Future studies are essential to address the durability of immune responses, the breath of protection against antigenically drifted strains, and comparative performance against existing intranasal and intramuscular vaccines. Taken together, the ability of PIV5 vectors to elicit both mucosal and systemic immune response combined with a safety profile, this platform represents a promising candidate for next-generation intranasal influenza vaccine development.

### 3.2 Non-replicating intranasal platforms

#### 3.2.1 Adenoviral vectors (NasoVAX)

NasoVAX is the most clinically advanced intranasal vaccine candidate based on a replication-deficient adenovirus serotype 5 (Ad5) vector designed to express influenza HA in the respiratory epithelial cells, thereby inducing both local mucosal and systemic immune responses in the absence of productive viral replication (112, 113).

Several features make adenoviral vectors particularly attractive for intranasal influenza vaccination. First, the Ad5 vector promotes high-level antigen expression in respiratory epithelial cells, which supports the development of a robust immune response (114). Second, several studies have suggested that intranasal delivery can overcome the impact of pre-existing anti-Ad5 immunity, which has limited the efficacy of parenterally administered Ad5-based vaccines, allowing effective immunogenicity in Ad5-seropositive individuals (113–115). And lastly, the replication-deficient, non-segmented nature of adenoviral vectors minimizes the risks associated with viral shedding and genetic reassortment (114).

Preclinical studies demonstrated that intranasal immunization with Ad5-vectored influenza vaccines elicits robust antigen-specific immune responses at both mucosal and systemic levels (116–119). In animal models, Ad5-vectored vaccine expressing influenza HA induced serum IgG, neutralizing antibodies, mucosal IgA, and T-cell responses characterized by IFN-γ production, supporting a balanced immune profile that may contribute to protection against influenza virus challenge (116–118).

Taken together, these favorable features supported the translation of Ad5-vectored influenza vaccines into clinical evaluation. On this basis, NasoVAX, an experimental recombinant, monovalent intranasal Ad5-based influenza vaccine, has advanced into phase II clinical studies (ClinicalTrials.gov NCT03232567) and currently represents the most clinically developed intranasal influenza vaccine of a non-replicating intranasal adenoviral platform, demonstrating favorable safety, tolerability, and immunogenicity in humans (114).

Healthy adults vaccinated with NasoVAX developed robust HA-specific serum IgG and neutralizing antibody responses comparable to those elicited by the licensed inactivated influenza virus vaccine Fluzone, while also inducing mucosal IgA responses, an immunological feature not typically observed following IM vaccination (114). Notably, HAI titers persisted for greater than 12 months following a single intranasal dose (114). In addition to humoral immune response, NasoVAX vaccination was associated with influenza virus-specific T-cell responses, characterized by IFN-γ production, highlighting the platform’s capacity to elicit both mucosal and systemic immune response (114).

Despite encouraging clinical findings, additional studies are required to better characterize the magnitude, durability, and breadth of immune responses elicited by NasoVAX, particularly across different age groups. Its ability to induce durable antibody responses together with mucosal IgA and influenza-specific T-cell responses highlights the potential of adenoviral vectors to overcome immunological gaps associated with conventional IM vaccination. As intranasal vaccine strategies continue to evolve, the clinical progress of NasoVAX underscores the potential of replication-deficient adenoviral vectors as a viable non-replicating intranasal strategy for next-generation influenza vaccines.

#### 3.2.2 Adjuvanted protein-based

Adjuvanted protein-based platforms represent a class of non-replicating intranasal influenza vaccines that rely on the mucosal delivery of purified, often recombinant, viral antigens formulated with immunostimulatory adjuvants to enhance local and systemic immune responses (120, 121).

Recombinant protein antigens can be produced using well-established expression systems, allowing precise antigen design, rapid strain updating, and the inclusion of conserved epitopes to broaden protective coverage (122). However, when administered intranasally, soluble protein antigens are typically poorly immunogenic due to limited uptake across the nasal epithelium and the tolerogenic mucosal environment, which favors antigen clearance and immune regulation rather than robust priming. To overcome this, intranasal protein-based influenza vaccines are formulated with adjuvants to enhance the immune response and vaccine efficacy (123, 124). A range of adjuvants have been used to enhance the immunogenicity of these vaccines, including bacterial toxin–derived adjuvants (*e.g.*, detoxified derivatives of cholera toxin), toll-like receptor (TLR) agonists such as CpG oligodeoxynucleotides, as well as nano- or polymer-based delivery systems designed to enhance antigen stability, epithelial uptake, and mucosal immune priming (125–128).

In animal challenge models, adjuvanted protein-based intranasal vaccines conferred protection against homologous viral strains and partial cross-protection against antigenically drifted variants (129, 130). Protective immunity was associated with robust serum IgG, HAI titers, neutralizing antibodies, and mucosal IgA in the URT (127, 129, 130). In addition, vaccination elicited influenza virus-specific CD4^+^ and CD8^+^ T-cell responses, including Th1-associated cytokine profiles, which support mucosal antibody production and cross-protective immunity (127, 129, 130).

Based on the preclinical performance, the first clinical evaluation has recently been reported. In a phase I trial of a nanoemulsion−adjuvanted recombinant influenza A/H5 hemagglutinin (NanoVax H5 + W805EC) vaccine (ClinicalTrials.gov NCT05397119), the intranasal formulation was well tolerated in healthy adults, with mild local reactogenicity and no serious adverse events (131). Importantly, vaccinated individuals developed mucosal IgA in the URT and serum IgG, alongside evidence of memory B−cell and influenza virus−specific CD4^+^ T−cell responses (131). Together, these results provide the first clinical evidence that intranasal adjuvanted recombinant protein vaccination can engage both mucosal and systemic compartments in humans, supporting further investigation of this platform as a strategy to improve protection against influenza viruses.

Despite encouraging findings, several challenges remain for the adjuvanted protein-based vaccine platform. The durability and magnitude of immune responses, particularly neutralizing antibodies, remain variable. Optimization of adjuvant formulation, antigen dose, and delivery methods will be critical to enhance immunogenicity while preserving an acceptable safety profile. Furthermore, the breadth of protection against antigenically drifted or emerging influenza strains in humans remains to be fully characterized. Addressing these gaps will be essential to determine the full potential of adjuvanted protein-based intranasal vaccines as a next-generation, non-replicating platform for broad, durable, and safe protection against influenza.

#### 3.2.3 Virus-like particle (VLP)

VLP are non-replicating, self-assembling nanoparticles that mimic the structural organization of authentic virions while lacking viral genetic material, making them safe and well suited for vaccine development (132). Their particulate and multivalent nature facilitates efficient B-cell receptor engagement and antigen uptake by antigen-presenting cells, thereby promoting both humoral and cellular activation without viral replication (133–135).

Preclinical studies have shown that intranasal influenza VLP vaccination provides robust protection against homologous virus challenge (136–139). These vaccines in selected formulations, have been shown to induce broad immune responses and partial protection against antigenically distinct influenza strains, particularly when incorporating conserved antigens, multivalent HA constructs, or mucosal adjuvants (136–138, 140, 141). Protective immunity was associated with a multifaceted immune response, including serum IgG and neutralizing antibodies, robust mucosal IgA in the URT, and influenza-specific CD4^+^ and CD8^+^ T-cell responses (136, 137, 139–141). Importantly, humoral immune responses correlated strongly with protection against homologous viral challenge (136, 138, 139), while cellular responses, particularly CD8^+^ T cells targeting conserved antigens, were critical for heterosubtypic and cross-protective immunity (137, 140, 141).

Although VLP-based vaccines demonstrate robust immunogenicity and protective efficacy in preclinical models, the clinical translation of this platform has been limited to IM administration (142, 143). This reflects several challenges, including antigen dose optimization, particle stability, and mucosal retention to ensure consistent immunogenicity following intranasal administration. In addition, the selection and safety evaluation of mucosal adjuvants remain critical, as historical concerns about intranasal adjuvant use require a careful balance between immune enhancement and local tolerability (144, 145).

From a safety perspective, the non-replicating and genome-free nature of VLPs represents a major advantage for intranasal delivery. The absence of viral replication reduces risks associated with shedding and reassortment, making VLPs an attractive option for populations in which replicating intranasal vaccines may be contraindicated (146). Furthermore, the VLP platforms allows the inclusion of conserved influenza antigens or immunostimulatory molecules, providing flexibility to tailor immune responses toward broader and more durable protection (132).

Looking forward, clinical studies are needed to determine whether the robust mucosal and cellular immune profiles observed in animal models can be translated to humans and capable of protecting against both seasonal and drifted influenza strains. If these challenges can be addressed, intranasal delivery of VLP vaccines may emerge as a versatile, safe, and broadly protective non-replicating strategy within the expanding landscape of next-generation influenza vaccines.

## 4 Discussion

Intranasal influenza vaccination represents a promising strategy to enhance protection against influenza virus infection and overcome the limitations of conventional IM vaccination by inducing both local and systemic immune responses. Across the diverse intranasal platforms under development, mucosal immunity, particularly secretory IgA and T- and B-cell immune responses, plays a central role in shaping protection against influenza virus infection (25, 38, 54). Preclinical and clinical studies consistently demonstrate that intranasal vaccination can elicit immune response profiles that differ from those induced by IM vaccines (6), with implications for viral control in the URT and, potentially, transmission.

LAIV remains the only licensed intranasal seasonal influenza vaccine and is efficacious, particularly in the pediatric population (76, 79, 84, 85, 87). However, its performance in adults has been more variable, reflecting the influence of host factors, such as pre-existing immunity, on vaccine virus replication and mucosal immune engagement (78, 91). In addition, strain-specific differences in viral fitness and clinical contraindications in certain populations further limit the consistency and broad applicability of replication-dependent intranasal vaccination strategies (67, 76, 92). Together, these challenges have driven the development of next-generation intranasal influenza vaccine platforms designed to preserve the immunological advantages of mucosal vaccination while improving consistency, safety, and applicability across diverse populations.

In this context, diverse next−generation intranasal influenza vaccine platforms have emerged, including replicating and non-replicating viral platforms. Although these approaches differ in design, they share the goal of eliciting robust mucosal and systemic immune responses while minimizing or eliminating vaccine virus replication in the URT. Replication-restricted platforms, such as M2SR and PIV5 vector-based vaccines, are designed to preserve robust antigen expression and mucosal immune activation while offering an improved safety profile by restricting replication or eliminating reassortment potential, thereby potentially expanding the applicability of intranasal vaccination across different populations (98, 101, 107, 110). In contrast, non-replicating platforms, including Ad5-vectored, adjuvanted recombinant protein, and VLP-based vaccines, prioritize safety, manufacturing flexibility, and antigen modularity, relying on optimized delivery systems and adjuvantation to achieve effective mucosal immunogenicity (123, 124, 132).

Despite these advances, important trade-offs remain across intranasal influenza vaccine platforms. Replicating platforms, including LAIV, M2SR and PIV5-based vectors, are effective at inducing robust mucosal IgA and cellular immune responses, reflecting their ability to mimic natural infection. This is likely one of the reasons they are often associated with broader immune responses and, in some cases, cross-protection. However, their performance can be less consistent in adults with pre-existing immunity, where prior exposure may limit viral replication and reduce antigen availability in the URT. In contrast, non-replicating platforms, including adenoviral vectors, adjuvanted protein-based and VLP, offer a safer profile and greater flexibility in antigen design, with fewer concerns related to viral shedding or reassortment. These platforms also have practical advantages in terms of scalability and the ability to rapidly update antigens in response to circulating strains. At the same time, inducing robust and durable mucosal immune response without replication remains challenging, and often depends on optimization of adjuvants and delivery strategies. Overall, these differences highlight that no single platform fully addresses all immunological and translational requirements, underscoring the need for continued platform-specific optimization and comparative evaluation.

An important next step is to better connect these immunological profiles with clinically meaningful outcomes. While HAI titers remain a well-established correlate of protection for IM vaccines, their relevance for intranasal approaches is more limited, particularly given the importance of immune responses at the site of viral entry. In this context, mucosal markers such as secretory IgA in the URT have emerged as an important and measurable biomarker for intranasal vaccines, typically assessed using nasal washes or swabs (147). Several studies suggest that higher mucosal IgA levels are associated with reduced viral shedding and, in some cases, protection against infection, although variability in sampling and assay standardization remains a challenge (38, 53, 148). Beyond antibody responses, virological endpoints such as viral load and duration of shedding in the URT provide a more direct measure of vaccine impact on viral replication and are particularly relevant for assessing effects on transmission (149, 150). Integrating these endpoints into clinical trial design, together with longitudinal sampling to capture the kinetics of mucosal responses, will be essential for establishing more relevant correlates of protection for intranasal influenza vaccines.

In addition to immunological considerations, the performance of intranasal vaccines is strongly influenced by delivery and formulation parameters. Factors such as dosing volume, formulation properties, such as viscosity and stability, and device design affect antigen distribution, retention and consistency of deposition within the nasal cavity (151–153). Mucociliary clearance further limits the duration of antigen exposure, potentially limiting effective immune priming (153, 154). Achieving reproducible delivery to relevant sites in the URT remains challenging, particularly across different age groups and anatomical variability (155, 156). These constraints are platform-dependent, with replicating and vector-based approaches relying on efficient infection of epithelial cells, whereas non-replicating protein and VLP-based vaccines depend more on formulation and adjuvants to enhance uptake and immunogenicity (157, 158). In this context, continued optimization of formulation stability, delivery devices, and dosing strategies will be essential to ensure consistent immunogenicity and clinical performance across intranasal vaccine platforms.

Importantly, several emerging intranasal vaccine platforms are actively addressing key formulation and delivery constraints associated with mucosal immunization. Advances in platform design, including viral vectors, VLPs, and optimized mucosal adjuvants, have demonstrated improved antigen stability, enhanced epithelial uptake, and prolonged mucosal retention, thereby promoting more consistent immunogenicity (151, 159–161). In parallel, formulation strategies such as nanoemulsions, dry powder delivery systems, and mucoadhesive carriers have been shown to enhance antigen residence time in the nasal cavity and mitigate the effects of mucociliary clearance (153, 162, 163). Moreover, efforts to improve thermostability and develop formulations less dependent on cold-chain infrastructure have demonstrated increased stability under variable temperature conditions (163, 164). Together, these advances indicate that next-generation intranasal vaccine platforms are increasingly addressing not only immunological requirements but also critical translational and logistical challenges associated with large-scale deployment.

Taken together, these advances reflect a broader shift in influenza vaccine development toward strategies that engage both mucosal and systemic immune responses. Rather than focusing solely on serum antibody titers, intranasal strategies increasingly highlight immune mechanisms at the site of viral entry, including secretory IgA and tissue-associated T- and B-cell responses. Evidence from both preclinical and clinical studies supports this conceptual transition, while also pointing to the need for improved immunological correlates of protection that better capture the contribution of mucosal immunity to viral control in the URT.

Further progress in this field will depend on integrating mechanistic insights from mucosal immunology with rigorously designed clinical studies across different age groups and risk populations. In particular, greater understanding is needed regarding the durability, breadth, and functional relevance of mucosal immune responses, as well as how these responses are shaped by prior influenza exposure. At the same time, safety considerations continue to influence the development of intranasal vaccines, particularly in light of concerns related to mucosal adjuvants and, for replicating platforms, the potential for viral shedding or reassortment. Current candidates increasingly address these risks through replication-restricted or non-replicating designs, improved adjuvant formulations, and routine clinical monitoring of local reactogenicity and viral shedding. Addressing these questions will be essential to defining the role of intranasal vaccines alongside existing influenza prevention strategies and to determining their potential to close persistent gaps in influenza control.
